# Development of an Encapsulation Method for Trapping the Active Materials from Sour Cherry Biowaste in Alginate Microcapsules

**DOI:** 10.3390/foods12010130

**Published:** 2022-12-27

**Authors:** İrem Toprakçı, Mehmet Torun, Selin Şahin

**Affiliations:** 1 Chemical Engineering Department, Faculty of Engineering, Istanbul University—Cerrahpasa, Avcilar, 34320 Istanbul, Turkey; 2 Food Engineering Department, Faculty of Engineering, Akdeniz University, 07058 Antalya, Turkey

**Keywords:** agrifood waste, natural antioxidants, green technology, ionic gelation, particle morphology

## Abstract

This study aims to contribute to those valorization approaches for the recovery process of high-value-added substances in environmentally friendly ways. In this study, one of the most consumed juice products was selected for providing waste byproducts (peel). Sour cherry peels were subjected to automatic solvent extraction using a GRAS solvent (aqueous 80% ethanol, *v*/*v*). Then, encapsulation for the preservation of the related extract was performed by ionic gelation in alginate beads. The process conditions (gelling medium concentration, wall material concentration, and hardening time) were optimized by a Box–Behnken design (statistical experimental design approach). An almost 80% encapsulation efficiency was achieved under the proposed method (7.8% CaCI_2_, 1.3% alginate, and 26 min). The inhibition effect of the produced capsules against DPPH (2,2-diphenyl-1-picrylhydrazil) radicals also shows that the current products might represent potential alternative natural antioxidants for food formulations. The morphological properties were also measured.

## 1. Introduction

The valorization of biowaste derived from agricultural and food processing is a very important study in terms of the circular economy since the related residues possess various beneficial phytochemicals that minimize environmental damage [1]. Sour cherry pomace is a rich source of natural antioxidants [2]. A total of 25% of the total weight remains as residual waste from wine and juice processing [3]. Therefore, an investigation for the recovery of downstream processing is of great value to the production of high-value-added substances. On the other hand, these fine chemicals have been reported to represent alternatives as preservatives in food and cosmetic formulations [4,5,6]. Sour cherry pomace has been known to have a significant number of phenolic substances [7]. The dried extract powder contained 91.29 mg of total phenolic substance per gram of powder as gallic acid based on the report of Çilek et al. [8]. The main phenolic compounds in the extract are anthocyanins, such as cyanidin glycosides [9]. Its antioxidant activity was also comparable to that of butylated hydroxyanisole (one of the most consumed synthetic additives) and ascorbic acid [10]. However, the stability of these compounds should be improved due to the degradation problems when exposed to external influences, such as heat, light, humidity, and the acidity of the medium [11]. 

Ionic gelation can be seen as an option for an encapsulation method due to its simplicity, efficiency, and low cost [12]. There is no need to use complex systems like freeze-drying methods. Unlike spray drying, there is also no high-temperature application [13]. This is very valuable in terms of preventing the decomposition of the phenolic components, which are especially heat-sensitive components, during processing. In the presence of CaCI_2_ (multivalent cations, such as Ca^++^), polysaccharide alginate (sodium alginate) forms crosslinks to produce an edible film around the bioactive core and form a polymer film [14].

Even though there are many studies on the recovery of bioactive substances from sour cherry peels [10,15,16], encapsulation studies for the preservation of the related extract is very scarce. Çilek et al. used a conventional extraction method to extract phenolic compounds from sour cherry peels by using aqueous ethanol [8]. Then, they dried the extract via a freeze-drying method before coating the active material with maltodextrin and gum Arabic. Šaponjac et al. and Petrović et al. applied freeze-drying to encapsulate sour cherry peel extract with whey and soy protein as the wall materials, respectively [7,17]. Furthermore, they applied the encapsulated sour cherry peel extract in cookie formulations, where the functional properties of the food products were enhanced. Luca et al. used gum Arabic and a maltodextrin mixture to trap the phenolics of the sour cherry [18]. They observed that encapsulation resulted in the prevention of active materials from the coating material since it behaved like a barrier. As far as we know, there has been no application of ionic gelation for the encapsulation of active materials in sour cherry peels. Actually, ionic gelation with alginate is a better way to encapsulate phenolic compounds, such as anthocyanins, due to their sensitivity to heat [19]. Depending on our earlier reports [20,21,22], automatic solvent extraction was used as an efficient extraction method for the recovery of active materials from the selected biowaste. There are some process parameters, such as coating material (alginate) and gelling medium (calcium chloride) concentrations, hardening time for the capsules, and the ratio of core materials to wall material in ionic gelation systems. Therefore, the effects of these parameters should be identified, and then the ionic gelation system should be optimized. In this study, the active material concentration was kept constant depending on the preliminary study. The concentrations of alginate and CaCI_2_ and the hardening time were chosen as the process parameters (factor) affecting the present ionic gelation system. As for the dependent variables, the antioxidant activity of the produced capsules and encapsulation efficiency (EE) with respect to total phenolic content (TPC) was selected. A Box—Behnken-type design under a response surface method (RSM) was employed for the statistical experimental design of the ionic gelation system of the active materials from sour cherry peels into alginate beads. The morphology and shape properties of the developed microbeads were also investigated via a stereo microscope and scanning electron microscopy (SEM) to evaluate the morphology. The alginate beads were also subjected to physicochemical analysis, such as water activity, bulk density, and moisture content. 

## 2. Materials and Methods

### 2.1. Materials

A commercial juice company (DİMES) located in İzmir (Turkey) supplied the sour cherry samples. The fruit samples were subjected to some physicochemical analyses, such as pH, total soluble solids content (°Brix), and total titratable acidity (g-malic acid/100 g). The total soluble solids content was measured at 20 °C using a digital refractometer (Atago PAL-1 BLT/i, Tokyo, Japan), while pH analyses were performed with a pH meter (Orion 4-Star pH meter, Thermo Scientific, Waltham, MA, USA). Titratable acidity was determined by potentiometric titration with 0.1 N NaOH up to pH 8.1 [23]. The total soluble solids content of the fruits was 13 °Brix. The pH of the samples was 3.2. Total titratable acidity was measured as 1.5 g-malic acid/100 g. The peels of the samples were separated by hand before the fresh peels were used in the extraction. 

Folin-Ciocalteu, DPPH (2,2-diphenyl-1-picrylhydrazil), trolox (6-hydroxy-2,5,7,8-tetramethylchroman-2-carboxylic acid), gallic acid, HCl, ethanol, methanol, Na_2_CO_3_, sodium alginate, and CaCI_2_ dihydrate were obtained from Sigma-Aldrich (St. Louis, MO, USA). 

### 2.2. Automatic Solvent Extraction

The extraction unit (Velp Scientifica, Usmate, Italy) was provided by the VELP^®^ society. The extraction process was performed with 80 mL of ethanol: water: 0.1 N HCI mixture (68:20:12, *v*/*v*/*v*). HCI was particularly added into the solvent since acidic solutions prevent the degradation of anthocyanin substances by balancing the anthocyanins in flavylium ion form [24]. The pH of the solution was ~3. The amount of raw material to be extracted was 1 g. Total extraction time was 92 min, including immersion time (20 min), washing time (40 min), recovery time (30 min), and cooling time (2 min) [25]. Briefly, the fresh samples were put in cellulose soxhlet extraction thimbles (33 mm × 80 mm). Then, the thimbles were placed in glass cups containing the solvent. Expectedly, the level of solvent decreased since the remaining was recovered in a collection tank. The extract filtered from the cellulose thimble was not subjected to filtration.

### 2.3. Encapsulation Method

Alginate-based ionic gelation was used as the encapsulation method. Wall material (alginate) concentration was adjusted to 10 g/L, 15 g/L, and 20 g/L, respectively. On the other hand, the gelling medium (CaCl_2_) concentration was adjusted to 20 g/L, 85 g/L, and 150 g/L, respectively. Then, the mixture of the core to the wall material (1/2, *v*/*v*) was mixed by vortex for 3 min. In total, almost 170 mL of extract and 340 mL of alginate solution were consumed. The mixture was added to the medium (250 mL CaCl_2_) on a magnetic stirrer. Then, the capsules were left to be mixed in the CaCl_2_ solution for several time periods (15, 30, and 45 min) at ambient conditions. The agitation of the magnetic stirrer was 275 rpm.

### 2.4. Shape and Morphology of the Alginate Microcapsules

The morphology of the capsules was performed by using a stereo microscope (Stemi 2000-C, Zeiss, Germany) connected to an AxioCam digital camera. The capsule size was determined by using the Zeiss (Carl Zeiss Microscopy GmbH, Jena, Germany) program. Sphericity factor (SF) and the roundness (Rn) values of the capsules were calculated according to Equations (1) and (2) [26]:

(1) $$\mathrm{Spherical}\;\mathrm{factor}\;\left(\mathrm{SF}\right)=\frac{d_{\max}-d_{\min}}{d_{\max}+d_{\min}}$$

(2) $$\mathrm{Roundness}\;\left(\mathrm{Rn}\right)=\frac{P^{2}}{4\pi A}$$

where d_max_ is the maximum diameter, d_min_ is the minimum diameter, P is the perimeter, and A is protection area of the capsules. 

Scanning electron microscopy (SEM) was used to determine the morphology of the capsules. The capsules were fixed onto the stubs of Zeiss LEO 1430 model SEM device. Coating treatment was applied with a thin layer of gold.

### 2.5. Physicochemical Analysis of the Alginate Microcapsules

Moisture content of the capsules was determined by drying the samples in an oven maintained at 70 °C for 24 h until a constant weight. Measurement of water activity was carried out by using a water activity meter (Testo 650 Water Activity System, Cole–Parmer, Vernon Hills, IL, USA). Additionally, the bulk density of the microcapsules was calculated by application of weight/volume ratio after approximately 2 g of powder was transferred to the 10 mL graduated cylinder [27].

### 2.6. Bioactivity Measurements

A total of 100 mg of microcapsules were dissolved in 3 mL of ethanol: acetic acid: water mixture (50:8:42, *v*/*v*/*v*). After mixing the mixture with a vortex for 1 min, the mixture was subjected to an ultrasonic bath at ambient conditions. Using the Folin–Ciocalteau method, 100 µL of the microcapsule mixture was taken into 2000 µL of Folin–Ciocalteau reagent (10%, *v*/*v*). After this mixture was kept in the dark for 5 min, 1800 µL of the sodium carbonate solution (7.5%, *w*/*v*) was added to the mixture and mixed. After the resulting mixture was kept in the dark for 1 h, the absorbance values were recorded at 765 nm in a UV spectrophotometer (PG Instruments, T60 / Leicestershire and England). Total phenolic content (TPC) was given in mg gallic acid equivalent (mg-GAE) per liter (ppm) [21].

In order to evaluate the efficiency of the microcapsules, the surface phenolic content (SPC) of the microcapsules was also calculated. A total of 100 mg of beads were dissolved in 3 mL ethanol-methanol solution (1:1, *v*/*v*) by keeping them in an ultrasonic bath (Protech, Istanbul, Turkey) for 5 min at ambient temperature [28]. The SPC results are also given in ppm units. 

In the case of antioxidant activity, in vitro DPPH assay was adopted [28]. A total of 15 mg of microcapsules were dissolved in 3 mL ethanol:acetic acid:water (50:8:42 *v*/*v*/*v*). After mixing the mixture with a vortex for 1 min, the mixture was subjected to an ultrasonic bath at 40 °C. DPPH solution was added into the mixture. Then, the samples were kept in the dark for 1 h. The measurements were read at 517 nm. The results were given in mg trolox equivalent per gram dried microcapsule (mg-TEAC/g-DM).

### 2.7. Encapsulation Efficiency

Encapsulation efficiency (EE) shows the success of the microencapsulation method with quantitative findings. It is expressed by the following equation:

(3) $$\mathrm{EE}\;(\%)=\frac{\mathrm{TPC}-\mathrm{SPC}}{\mathrm{TPC}}$$

### 2.8. Experimental Design

Response surface approach was utilized to determine the impacts of the ionic gelation factors (A, B, and C) with their interactions (AB, AC, and BC) and quadratic (A^2^, B^2^, and C^2^) terms, as well as performing the modeling and optimization studies. Box–Behnken design was selected as the subtype of the RSM. While encapsulation efficiency (Y_1_) and antioxidant activity (Y_2_) were the responses, the CaCI_2_ concentration (A), alginate concentration (B), and hardening time (C) were ionic-gelation-process parameters. The relationship between A, B, and C was formed to estimate Y_1_ and Y_2_. Design-Expert software (12.0.1.0) was used for the application of the RSM. Table 1 demonstrates the process factors, their levels, and the codes for the experimental design of the microencapsulation of active substances in sour cherry peels. 

## 3. Results and Discussions

### 3.1. Microencapsulation of the Active Substances from the Sour Cherry Peels in the Alginate Microcapsules

Design-Expert software produced 17 experimental values with several combinations of process parameters, depending on the design type (Box–Behnken) of the RSM (Table 2). An encapsulation efficiency of ≈80% was accomplished by the proposed method. On the other hand, the free radical scavenging activity against the DPPH radical varied from 2.50 to 3.26 mg-TEAC/g-DM. Luca et al. reported 86.07–98.29 % EE for the encapsulation of the same extract with a freeze-drying method using different wall materials (gum Arabic and maltodextrin mixture) [29]. When soy protein and whey were used in freeze-drying encapsulation method, the yields of each wall material were 94.9% and 90.1%, respectively [30]. It was also reported that EE with a 1/10 core to wall material changed between 69.38% and 77.83% [8]. Then, EE varied from 78.80% to 92.26% if the ratio was adjusted to 1/20. Oancea et al. encapsulated the sour cherry peel extract in whey proteins isolate with lower yield (almost 70% EE) [31]. Actually, it seems that the yield of the encapsulation depends on many factors such as the encapsulation method, wall material type, and the ratio of active material to wall material. As a simple, efficient and inexpensive method, ionic gelation might be a good option with the proposed yields [12]. 

### 3.2. Effects of the Ionic Gelation Parameters

**Figure 1:**
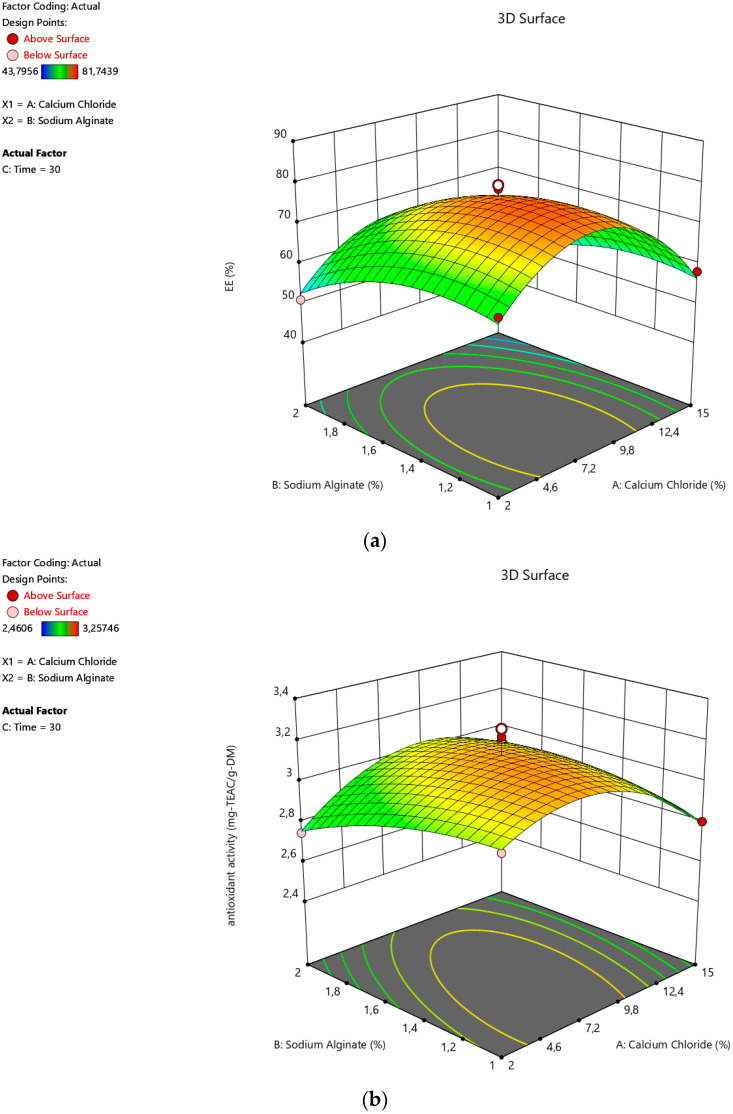
Effects of calcium chloride concentration to alginate concentration on the (**a**) encapsulation efficiency and (**b**) antioxidant activity of the alginate microcapsules containing the active substances from sour cherry peel extract.

**Figure 2:**
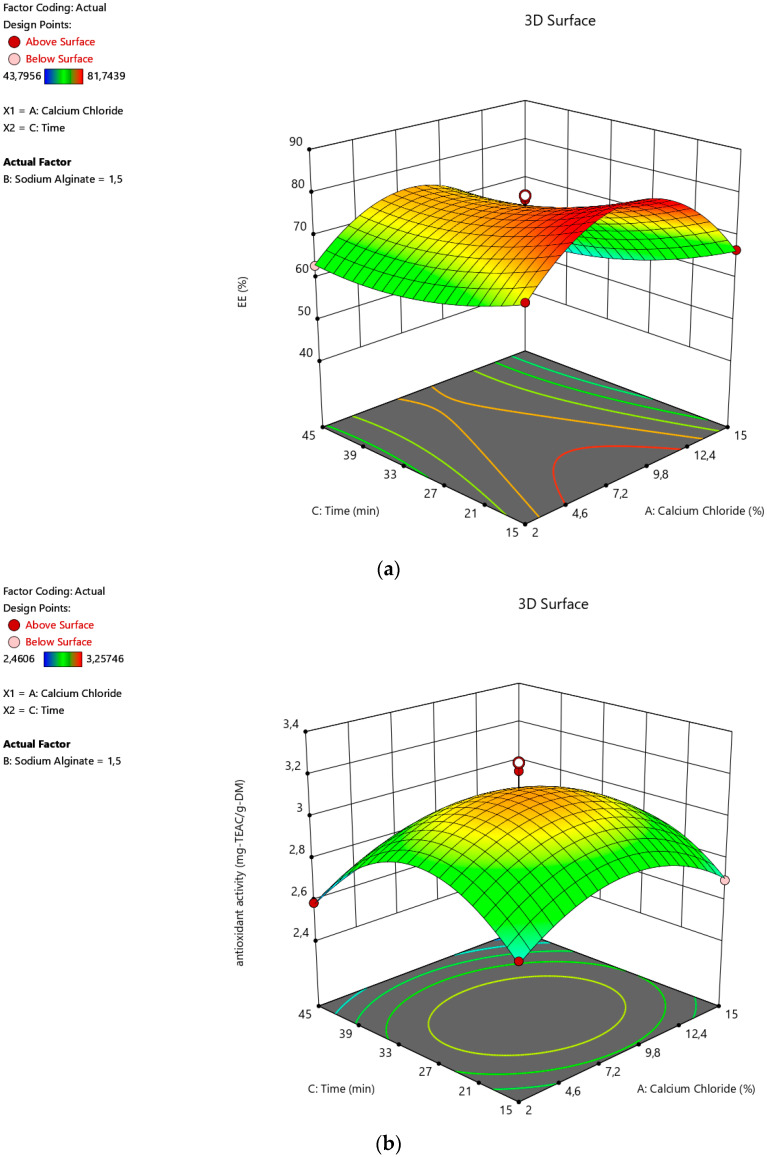
Effects of calcium chloride concentration with time on the (**a**) encapsulation efficiency and (**b**) antioxidant activity of the alginate microcapsules containing the active substances from sour cherry peel extract.

**Figure 3:**
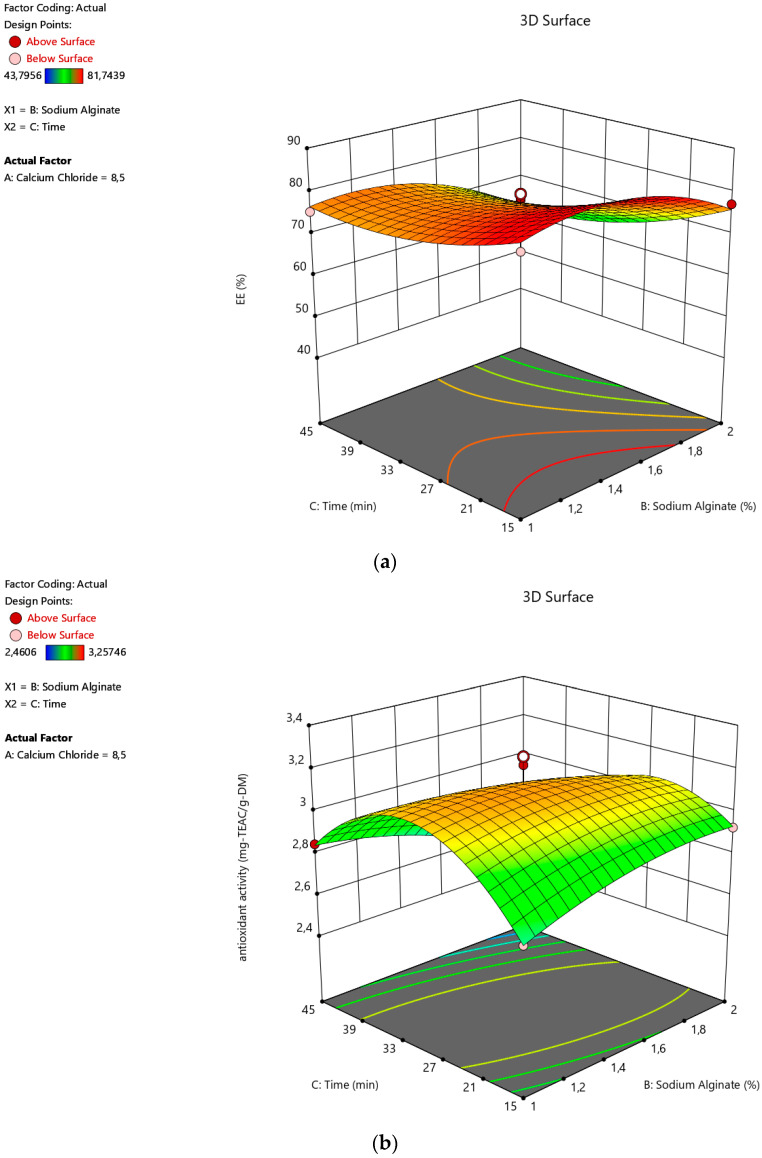
Effects of alginate concentration with time on the (**a**) encapsulation efficiency and (**b**) antioxidant activity of the alginate microcapsules containing active the substances from sour cherry peel extract.

Three-dimensional (3D) visuals can also be used to visually present the effect of the process parameters. These 3D images (Figure 1, Figure 2 and Figure 3) were produced with Design-Expert software. As seen in Figure 1a,b, the EE and antioxidant activity of the microcapsules increased sharply with CaCI_2_ concentration at first due to the fact that the Ca^++^ ions are necessary to form the capsules. The strength of the gel is also enhanced by increasing the concentration [32]. However, both of the responses reduced visibly after around a 10% concentration for CaCI_2_ (Figure 2a,b). A similar tendency of the gelling medium effect was also reported by Yousefi et al. during the encapsulation of *Viola odorata* Linn. extract by ionic gelation [33]. According to Halder et al. [34], the bivalent Ca⁺⁺ ions of the gelling-medium transferred to the beads, leading to the substitution of the active substance by the Ca⁺⁺ ions. Therefore, the yields of the encapsulation dropped by a higher degree for the CaCI_2_ concentration. Alginate concentration also favored encapsulation at first. Then, it started to decrease, as seen in Figure 1. This outcome is in agreement with the earlier reports on the alginate-based ionic gelation of several active materials [33,35,36,37]. Zam et al. explained this finding, with the increase in viscosity as the major reason for encapsulation efficiency [35]. Niizawa et al. also observed the same alginate effect on the encapsulation of astaxanthin in alginate microcapsules [38]. This was attributed to the occupation of the free spaces in the capsule, with excess alginate instead of the active material of the extract.

When the time for hardening is considered, EE was not significantly influenced, as seen in Figure 2a and Figure 3a. Additionally, the antioxidant activity of the capsules increased with time until ~30 min, as seen in Figure 2b and Figure 3b. Then, the activity against the free radicals began to drop with time. This is most probably because of the fact that the diffusion of the polar phenolic antioxidants into the polar gelling medium takes place due to the overexposure to the medium [39]. Similar time impacts were also observed during the encapsulation of cumin seed essential oil in alginate beads [27] and the encapsulation of yerba mate extract in alginate/chitosan microcapsules [40], respectively.

### 3.3. Modeling Study

**Figure 4:**
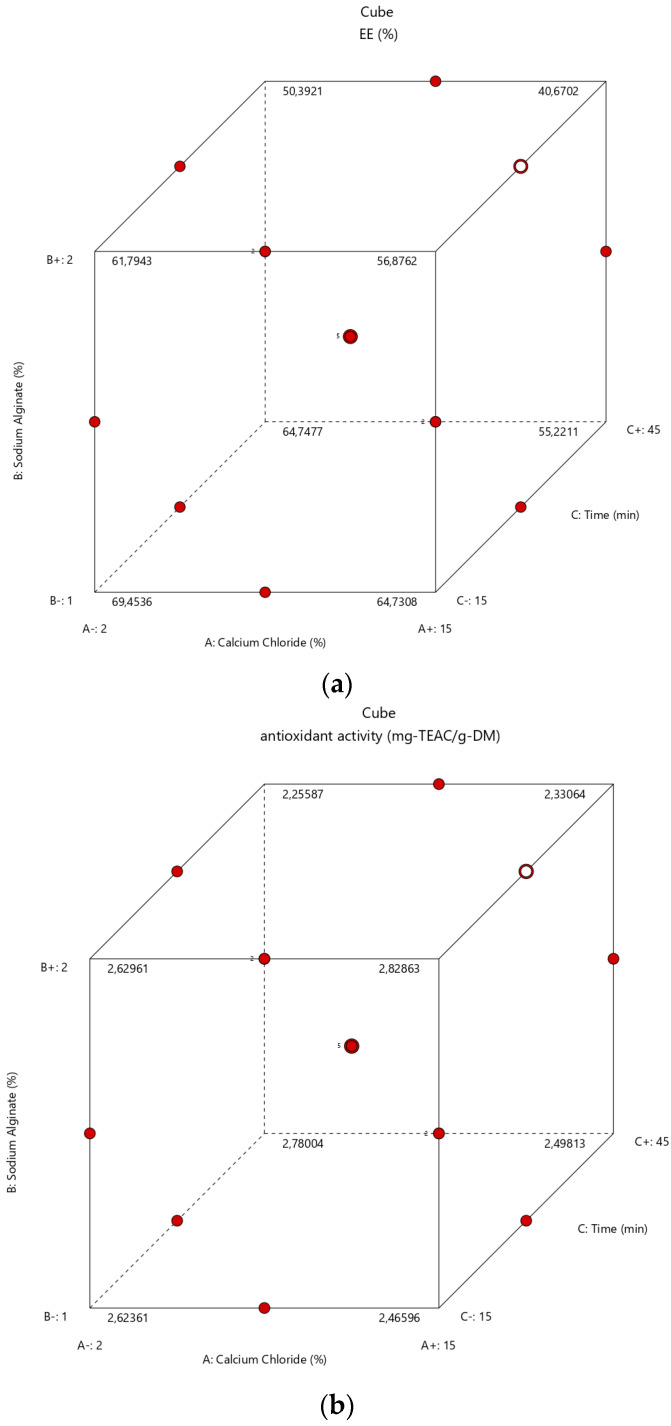
Illustration of Box–Behnken design for the three factors and three levels of the ionic gelation process for response EE (**a**) and reponse antioxidant activity (**b**).

The Box–Behnken design (as an independent quadratic design approach) was adopted for the present ionic gelation system. Since there are three factors with three levels, the Box–Behnken design was preferred. A total of 17 experimental runs with five replications at the center points (8.5% calcium chloride, 1.5% sodium alginate, and 30 min) were generated by the Design-Expert software. Figure 4 illustrates the classical quadratic designs for the current three factors of EE (Figure 4a) and antioxidant activity (Figure 4b), respectively.

The experimental data were subjected to the given quadratic model (Equation (4)):

(4) $$Y=\beta_{o}+\sum_{i=1}^{k}\beta_{i}X_{i}+\sum_{i=1}^{k}\beta_{ii}\;X_{i}^{2}+\sum \underset{i<j}{\sum }\beta_{ij}X_{i}X_{j}+\varepsilon_{i}$$

Y represents the response variable (EE and antioxidant activity), while k is the number of independent factors (three in this process). βₒ is the constant, whereas βᵢ, βᵢᵢ, and β_ij_ are the linear, quadratic, and interaction effects of the factors, respectively. 

The derived second order models (depending on Equation (4)) are given by Equations (5) and (6):

(5) $$Y_{1}=76.76-3.61\;A+5.55\;B-5.23\;C-0.0488\;\mathrm{AB}-1.20\;\mathrm{AC}-1.67\;\mathrm{BC}-16.54{\;A}^{2}-5.75\;B^{2}+3.50\;C^{2}$$

(6) $$Y_{2}=3.13-0.0207\;A-0.0404\;B-0.0854\;C+0.0892\;\mathrm{AB}-0.0311\;\mathrm{AC}-0.1325\;\mathrm{BC}-0.2025{\;A}^{2}-0.0603\;B^{2}-0.3135\;C^{2}$$

ANOVA (analysis of variance) was used to analyze the models statistically. Table 3 and Table 4 are the ANOVA findings with a significance level of 5% for Y_1_ and Y_2_, respectively. A model F-value of 24.59 means that the model is significant (Table 3), whereas a second model F-value of 14.67 implies the related model is significant, as seen in Table 4. On the other hand, a *p*-value < 0.05 denotes the significance of the model terms. In this instance, all the selected parameters and their second-order terms (A, B, C, A², B², and C²) are found to be statistically significant, as seen in Table 3. Table 4 indicates that C, BC, A², and C² are significant at *p* < 0.05 in the case of Y_2_. When the other model-fit statistics, such as the F-value, *p*-value, coefficient of variance (CV), coefficient of determination (R^2^), adjusted R^2^, predicted R^2^, and lack of fit, are considered, both of the models seem satisfactory for the representation of the experimental finding. Lack-of-fit F-values of 0.65 and 0.04 imply the lack of fit values for both responses (Y_1_ and Y_2_) are not significant. CV values (4.36% and 2.78%) of less than 10% are desirable since CV represents the dispersion of the data around the mean [41]. So, the smaller the CV, the less variation around the mean. Furthermore, the predicted R² of 0.8065 is in compliance with the adjusted R² of 0.9299 (Table 3), whilst the predicted R² of 0.9020 is in conformity with the adjusted R² of 0.8849 (Table 4). It is desirable that the difference between the predicted R^2^ and adjusted R^2^ be less than 0.2. 

### 3.4. Optimization and Validation Studies

**Figure 5:**
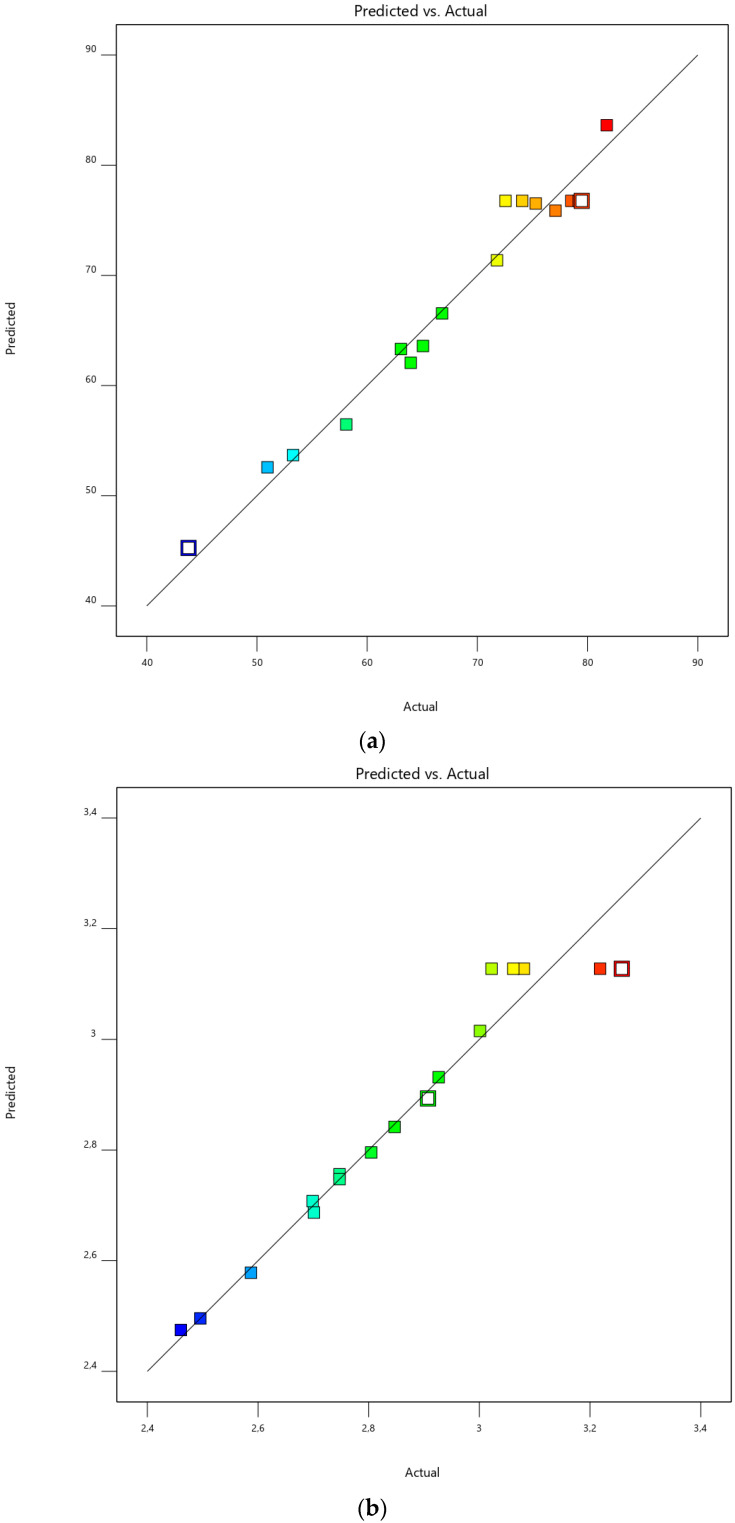
Relationship between the actual and predicted data under optimal conditions (7.8% CaCI_2_, 1.3% alginate, and 26 min) for response EE (**a**) and reponse antioxidant activity (**b**).

An optimization study was performed under the ranges of each process parameter to achieve the maximum EE and antioxidant activity. Table 5 gives the optimum conditions with the maximum yields, with a 0.89 desirability. In order to verify the predicted conditions, experimental studies under the optimum conditions (7.8% CaCI_2_, 1.3% alginate, and 26 min) were performed. The difference between the actual and predicted EE and antioxidant values (less than 2%) shows the reliability of the outcome of this study. Figure 5a (EE) and Figure 5b (antioxidant activity) also demonstrate the reliability of the produced models when less dispersion around the regression line is considered. 

### 3.5. Morphology and Size of the Alginate Microcapsules

**Figure 6:**
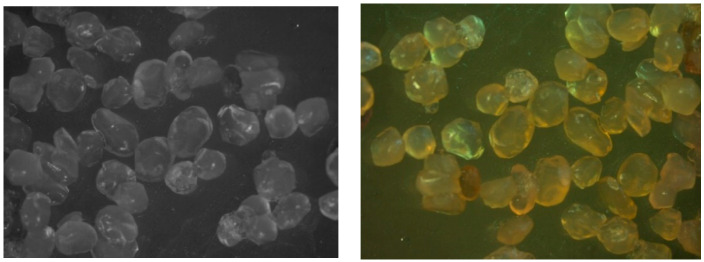
Microphotograph of the alginate microcapsules.

As spherical beads with a uniform size distribution are preferred for the delivery of active substances, the size and shape of the beads represent important parameters for food and pharmaceutical products [42]. The SF value was calculated via the ratio of the minimum capsule diameter to the maximum capsule diameter. This is mostly used for the teardrop and pear-shaped particles. While the SF value calculated by the ratio of the minimum and maximum diameters (to each other) is expected to be close to zero in a perfect sphere, it is also acceptable to be less than 0.05. Frequently, the Rn value indicates the spherical and elliptical characteristics of the particles. Although the Rn and SF values show how perfect the sphericity is, the Rn value is expected to be close to 1, contrary to the previously mentioned SF value [26]. The images of the alginate beads, including those from the sour cherry peel extract, are shown in Figure 6. The SF and Rn values were determined as 0.047 ± 0.017 and 1.126 ± 0.023, respectively. It is possible to say that the capsules are in an acceptable shape depending on the SF and Rn findings.

**Figure 7:**
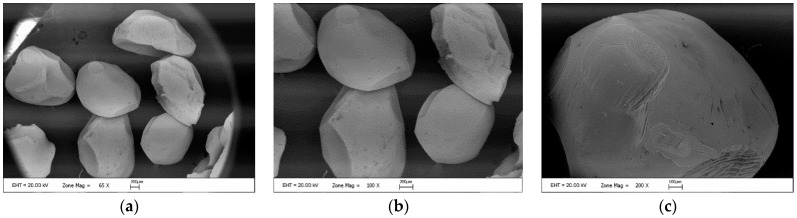
Scanning electron microscopy images of the alginate microcapsule with a different scale as ×65 (**a**), ×100 (**b**) and ×200 (**c**).

As seen in Figure 7, the SEM images support the previous size findings. The capsules are smooth and homogeneous because of the bond between the alginate and phenolic substances [43].

### 3.6. Moisture Content, Water Activity, and Bulk Density of the Alginate Microcapsules

The microcapsules produced under optimum conditions (7.8% CaCI_2_, 1.3% alginate, and 26 min) were subjected to physicochemical analyses, such as moisture content, water activity, and bulk density. The moisture content and bulk density of the beads were 52.30 ± 3.01% and 0.54 ± 0.07 g/mL, respectively. The water activity of the samples was determined to be 0.684 ± 0.02. Gholamian et al. mentioned that the high water-absorbing capacity of alginate gave rise to the formation of a 3D network with high moisture content [27]. Additionally, the macropores of the alginate matrix caused a low bulk density for the hydrogels. On the other hand, the hydrophilic or lipophilic character of the core material influenced the moisture content and density of the hydrogels.

## 4. Conclusions

The residuals from one of the most consumed juices were investigated in terms of the valorization of a rich source of natural antioxidants. In order to preserve the active material from many factors, an ionic gelation method was proposed using alginate microcapsules. A Box–Behnken design for the response surface method was adopted to develop a microencapsulation method for trapping the bioactive materials in the extract after the sour cherry peels were subjected to a green, environmentally friendly extraction method (automatic solvent extraction with aqueous ethanol). The optimum conditions (7.8% CaCI_2_, 1.3% alginate, and 26 min) were verified with a less than 2% error (the difference between the actual and predicted response values). All the fitting statistics (F-value (24.59 and 14.67), *p*-value (<0.05), lack of fit (>0.05), CV (<10%), R^2^ (>0.94) adjusted R^2^ (>0.88), and predicted R^2^ (>0.80)) denoted the satisfactory model equations for the microencapsulation of sour cherry peel extract in alginate beads. As a result of the increasing importance of functional foods recently, the microencapsulation process has gained more meaning for the food industry. Considering that the current samples have antioxidant activity, these products might find application as a potential food additive. In this way, the physical properties of the related food product might be improved by preventing the possible bitter taste of the extracts from being used directly and the release of undesirable colors into the food. Moreover, the capsules are in an acceptable shape (smooth and homogeneous), considering that regular size dispersion is preferred for the transportation of active substances in food applications.

## Figures and Tables

**Table 1:** Factors for the experimental design of the microencapsulation of the active substances in the sour cherry peels.

Process Parameter	Unit	Symbol	Coded Level		
			−1	0	1
Calcium chloride	%, *w*/*v*	A	2	8.5	15
Sodium alginate	%, *w*/*v*	B	1	1.5	2
Time	min	C	15	30	45

**Table 2:** Experimental results for the microencapsulation of active substances in sour cherry peels according to Box-Behnken design of RSM.

Run	A: Calcium Chloride (%, *w*/*v*)	B: Sodium Alginate (%, *w*/*v*)	C: Time (min)	TPC (ppm)	SPC (ppm)	EE (%)	Antioxidant Activity (mg-TEAC/g-DM)
1	2	1.5	45	7.93 ± 0.001	2.93 ± 0.001	63.06	2.59 ± 0.004
2	8.5	15	30	11.50 ± 0.002	2.39 ± 0.002	79.19	3.06 ± 0.001
3	8.5	2	15	8.11 ± 0.001	1.86 ± 0.001	77.09	2.93 ± 0.001
4	8.5	1.5	30	11.14 ± 0.005	2.39 ± 0.003	78.53	3.08 ± 0.003
5	8.5	1.5	30	10.79 ± 0.003	2.21 ± 0.001	79.47	3.26 ± 0.004
6	2	1.5	15	7.21 ± 0.004	2.04 ± 0.002	71.78	2.70 ± 0.001
7	8.5	2	45	6.14 ± 0.002	2.21 ± 0.003	63.95	2.50 ± 0.006
8	2	2	30	5.61 ± 0.001	2.75 ± 0.001	50.96	2.75 ± 0.001
9	15	2	30	4.89 ± 0.001	2.75 ± 0.002	43.80	2.91 ± 0.005
10	8.5	1	15	13.11 ± 0.004	2.39 ± 0.004	81.74	2.75 ± 0.005
11	15	1.5	45	4.36 ± 0.001	2.04 ± 0.003	53.28	2.46 ± 0.006
12	8.5	1.5	30	10.61 ± 0.006	2.75 ± 0.002	74.07	3.02 ± 0.001
13	15	1	30	11.68 ± 0.001	4.89 ± 0.001	58.10	2.80 ± 0.001
14	8.5	1.5	30	11.32 ± 0.006	3.11 ± 0.002	72.56	3.22 ± 0.004
15	8.5	1	45	11.86 ± 0.004	2.93 ± 0.001	75.30	2.85 ± 0.004
16	2	1	30	10.43 ± 0.004	3.64 ± 0.006	65.07	3.00 ± 0.001
17	15	1.5	15	8.82 ± 0.005	2.93 ± 0.001	66.80	2.70 ± 0.003

Data are given as the arithmetic mean of the three replicates.

**Table 3:** ANOVA test for encapsulation efficiency.

Source	Sum of Squares	df	Mean Square	F-Value	*p*-Value	
Model	1944.66	9	216.07	24.59	0.0002	significant
A-Calcium chloride	104.32	1	104.32	11.87	0.0108	
B-Sodium alginate	246.65	1	246.65	28.07	0.0011	
C-Time	218.65	1	218.65	24.89	0.0016	
AB	0.0095	1	0.0095	0.0011	0.9746	
AC	5.77	1	5.77	0.6567	0.4444	
BC	11.21	1	11.21	1.28	0.2959	
A²	1151.47	1	1151.47	131.06	<0.0001	
B²	139.00	1	139.00	15.82	0.0053	
C²	51.72	1	51.72	5.89	0.0457	
Residual	61.50	7	8.79			
Lack of Fit	20.23	3	6.74	0.6534	0.6215	not significant
Pure Error	41.27	4	10.32			
Cor Total	2006.16	16				
C.V.: 4.36%	R^2^ = 0.9693		Adjusted R^2^ = 0.9299		Predicted R^2^ = 0.8065	

**Table 4:** ANOVA test for antioxidant activity.

Source	Sum of Squares	df	Mean Square	F-Value	*p*-Value	
Model	0.8305	9	0.0923	14.67	0.0009	significant
A-Calcium chloride	0.0034	1	0.0034	0.5462	0.4839	
B-Sodium alginate	0.0130	1	0.0130	2.07	0.1931	
C-Time	0.0583	1	0.0583	9.28	0.0187	
AB	0.0318	1	0.0318	5.06	0.0593	
AC	0.0039	1	0.0039	0.6137	0.4591	
BC	0.0703	1	0.0703	11.17	0.0124	
A²	0.1726	1	0.1726	27.45	0.0012	
B²	0.0153	1	0.0153	2.44	0.1624	
C²	0.4137	1	0.4137	65.79	<0.0001	
Residual	0.0440	7	0.0063			
Lack of Fit	0.0012	3	0.0004	0.0364	0.9893	not significant
Pure Error	0.0429	4	0.0107			
Cor Total	0.8746	16				
C.V.: 2.78%	R^2^ = 0.9497		Adjusted R^2^ = 0.8849		Predicted R^2^ = 0.9020	

**Table 5:** Optimum conditions (desirability = 0.89) with the maximum predicted and experimental yields.

A (%, *w*/*v*)	B (%, *w*/*v*)	C (min)	Response	Predicted	Experimental	Error (%)
7.836	1.332	26.304	EE	79.507	78.125	1.74
			antioxidant activity	3.128	3.079	1.57

## Data Availability

Data are accessible on demand from the corresponding author. Code availability (software application or custom code)-Design-Expert software (12.0.1.0).
